# Incidence of Low‐energy Upper Extremity Fractures and the Risk Factors in Chinese People 50 years or Older

**DOI:** 10.1111/os.12448

**Published:** 2019-04-01

**Authors:** Xiao‐juan Zhang, Yan‐bin Zhu, Song Liu, Wei Chen, Bo Liu, Fei Zhang, Hong‐zhi Lv, Chen‐ni Ji, Xiao‐lin Zhang, Ying‐ze Zhang

**Affiliations:** ^1^ Department of Endocrinology The Third Hospital of Hebei Medical University Shijiazhuang China; ^2^ Department of Orthopaedic Surgery The Third Hospital of Hebei Medical University Shijiazhuang China; ^3^ Key Laboratory of Biomechanics of Hebei Province Shijiazhuang China; ^4^ Department of Statistics and Epidemiology Hebei Medical University Shijiazhuang China; ^5^ Chinese Academy of Engineering Beijing China

**Keywords:** Epidemiology, Low‐energy fracture, Mid‐ and elderly‐aged, Upper extremity

## Abstract

**Objective:**

To investigate the incidence of low‐energy upper extremity fractures and identify the associated risk factors in Chinese people aged 50 years or older.

**Methods:**

This study was a part of the Chinese National Fracture Survey, which was performed between January and May 2015 and aimed to investigate the epidemiology of traumatic fractures in China in 2014. The China National Fracture Study (CNFS) was registered with the Chinese Clinical Trial Registry (number ChiCTR‐EPR‐15005878). A stratified multistage cluster randomized sampling method was used to recruit subjects and the survey was conducted through a questionnaire. The relevant results have been published elsewhere. In the current study, 154 099 Chinese men and women aged 50 years or older were included for data collection and analysis. Low‐energy fractures were defined as fractures that were caused by simple falls from standing height. Individuals who had low‐energy upper extremity fractures were included in the case group and the remainder were included in the control group. Univariate and multivariate logistics regression analysis models were constructed to investigate the independent risk factors, after adjustment for confounding variables.

**Results:**

In total, 184 patients sustained low‐energy upper extremity fractures in 2014, indicating that the overall incidence was 119.4/100 000 persons, with 57.4 and 180.9/100 000 person‐years in men and women. Approximately 80% of fractures occurred at home and on the common road (other than high way). In men, alcohol consumption (OR, 2.12; 95%CI, 1.11–4.06), residence at ≥2nd floor without an elevator (OR, 2.86; 95%CI, 1.16–7.06), sleep duration<7 h/day (OR, 2.77; 95%CI, 1.42–5.37), and history of past fractures (OR, 3.10; 95%CI, 1.21–7.93) were identified as significant risk factors. In women, obesity (BMI ≥ 28.0) (OR, 1.86; 95%CI, 1.31–2.66), living in the central region in China (OR, 1.53; 95%CI, 1.01–2.31), living at a higher latitude (40°–49.9°N) (OR, 1.79; 95%CI, 1.02–3.14), alcohol consumption (OR, 2.40; 95%CI, 1.58–3.63), more births (OR, 1.45; 95%CI, 1.15–1.83), sleep duration <7 h/day (OR, 2.21; 95%CI, 1.53–3.20), and history of past fracture (OR, 2.70; 95%CI, 1.52–4.80) were identified as significant risk factors.

**Conclusion:**

Based on these results, health policies that focus on decreasing alcohol consumption and encouraging individuals to improve their quality and duration of sleep should be implemented in China. The significance of moving to a ground floor or to a building equipped with an elevator for men, and maintaining a healthy body weight for women should be emphasized to prevent upper extremity fractures.

## Introduction

Bone mass declines and the risk of fractures increases as people age, especially as women pass through menopause^1^. In fact, across their lifetime, osteoporosis‐related fracture risk in Caucasian women is approximately 40%^2^, ^3^, and in men is approximately 15%^4^. In 2001, the NIH Consensus Statement revised the definition of osteoporosis to include qualitative parameters related to low‐energy fractures and set fracture prevention as the primary treatment goal for patients with osteoporosis^5^. Over 60% of the overall fractures were related to osteoporosis and falls, and one‐third of them were upper extremity fractures, predominantly proximal humerus and distal radius fractures^6^, ^7^. Upper extremity fractures frequently lead to decreased daily living ability and impose a substantial burden on family and society, due to the higher incidence of morbidity and mortality, and the substantial costs involved^8^. Moreover, older adults who have osteoporosis‐related upper extremity fractures are at increased risk of future secondary falls, fractures, and even death^9^.

In the past two decades, Chinese population aging has been well‐documented, and in 2015 the average life expectancy reached 76.1 years. Data from National Bureau of Statistics of China showed that the number of middle‐aged and elderly individuals above 50 years was over 280 million by the end of 2016^10^. Annually, approximately 600 000 individuals had at least one upper extremity fracture^6^. This figure is expected to increase dramatically in the next few decades and, accordingly, the challenge is enormous for Chinese health policy‐making institutions.

Knowledge of population‐based epidemiologic characteristics of osteoporosis‐related fractures is fundamental to developing targeted public health programs. Up to now, most epidemiologic reference data used by Chinese studies or policy‐making institutions has been sourced from foreign research. However, these results might not be applicable to Chinese populations, due to the great difference in ethnic origins, economic development, cultural practices, and health‐care systems among countries. In addition, most of these foreign studies assessed upper extremity fractures using data from a single hospital or several hospitals in a region or focused on populations of a certain subgroup^11^, ^12^, ^13^, often with diverse incidence rates and controversial results regarding risk factors. Although several previous Chinese studies have reported the incidence of these fractures and associated risk factors^14^, ^15^, results might be compromised by small sample sizes or restricted geographic areas. Currently, data from national epidemiological surveys for upper extremity fractures remain scarce, for China and other countries.

Therefore, in conducting this study we aimed to: (i) report the national population‐based incidence rate of low‐energy upper extremity fractures for overall populations and for different subgroups stratified by age, gender, and site; and (ii) investigate the associated risk factors in terms of demographics, socioeconomics, geographical location, and individual lifestyle.

## Methods and Materials

### *Subjects*

This study was a part of the China National Fracture Study (CNFS, Chinese Clinical Trial Registry number ChiCTR‐EPR‐15005878). CNFS was a cross‐sectional questionnaire survey, carried out between January and May 2015, to investigate the population‐based epidemiology of traumatic fractures through 2014. Details of sampling methods and participant inclusion were described elsewhere^6^. A stratified multistage cluster randomized sampling method was used to recruit subjects. During the first phase, using the stratified random sampling method, we selected eight provinces (municipalities), with three in eastern, two in central and three in western regions, based on geographic location, climate, population size and socioeconomic development. During the second phase, within each targeted province (municipalities), sampling was done separately in urban and rural areas, using the probability proportional to size method. In each neighborhood or village, the households were calculated and selected, based on our preset sampling proportion. All members of eligible families that were invited to participate in this study had to have lived in their current residence for at least 6 months.

### *Standardized Questionnaires*

Standardized questionnaires were administered by our trained research team members for data collection. Written informed consent was obtained from each participant before data collection. Data on demographics, geographical conditions, socioeconomics, and individual lifestyles were documented. Fracture occurrence between 1 January and 31 December 2014 was self‐reported initially by participants and further confirmed by their providing clinical or radiographic data. When such medical data were unavailable, the survey team paid to obtain a new radiograph of their reported fracture site at a local hospital. Eight quality control teams with one for each province were responsible for checking random questionnaires (approximately 10% of all questionnaires) for potential omissions and errors. The CNFS was approved by the Institutional Review Board of the Third Hospital of Hebei Medical University.

### *Data Collection*

A low‐energy fracture was defined as a fracture that was caused by a slip, a trip or a fall from standing height. Fractures caused by high‐energy injuries (e.g. traffic trauma, fall from height, crushing injury, sharp trauma, and others) were excluded. Pathological fractures or metastatic fractures were also excluded. A total of 154 099 women and men aged 50 years and older participated in this study and 937 participants had at least one fracture of any site caused by either low‐energy or high‐energy injury in 2014. A total of 184 participants (case group) reported 185 cases of low‐energy fractures of humerus and radius and ulna. The remaining 153 162 participants without any fracture were defined as the control group.

### *Variables of Interest*

Variables of interest included age, height, weight and accordingly calculated body mass index (BMI), living areas, regions, latitude zone, ethnic origins, occupation, educational level, frequency of drinking intake, smoking status, alcohol consumption, dwelling place, sleep duration/day, history of past fracture, living situation (alone or with others), supplementation of calcium or Vitamin D or both for women and men, and age of menopause and number of extra births for women.

Specifically, the BMI was grouped based on the reference criteria suited to Chinese populations: underweight, <18.5 kg/m^2^; normal, 18.5–23.9 kg/m^2^; overweight, 24–27.9 kg/m^2^; obese, >=28 kg/m^2^. Current smoking and alcohol consumption were defined as positive (yes) if participants smoked >1 time/week or drank >1 time/month during the 2014 year or the past year before fracture occurrence. Similarly, within the timeframe, the average intake frequency of alcohol was provided by participants, and supplementation of calcium or Vitamin D or both was defined as positive (yes) if participants acknowledged that they take these medications for at least 1 month; otherwise, as negative (no).

### *Statistical Analysis*

Incidence rates of low‐energy upper extremity fractures were estimated for the overall population and for subgroups by age (5‐year interval), as well as by demographic factors such as ethnic origin, geographical region, education, occupation, and residency category, stratified by gender. Differences in incidence between categories of nominal variables, such as ethnic origin, regions, occupation, and residency category, were tested using the χ^2^‐test. Trends of incidence rates by age and education were tested in a univariate logistic regression model, by including these ordered categorical variables as a continuous variable. We also assessed the incidence rates of low‐energy humerus or radius (ulna) fractures based on sites (proximal, shaft, and distal), in men or women or men and women combined.

Two separate design‐based multiple logistic regression models were constructed to explore the risk factors associated with low‐energy upper extremity fractures among women and men. The abovementioned variables were all entered into the multivariate model. A stepwise backward‐elimination approach was used to exclude confounding covariates. Covariates were retained in the final model if the *P‐*value was ≤0.10. The odds ratio (OR) and the 95% confidence interval (95% CI) were used to indicate the correlation magnitude between variables and low‐energy fracture risk. The Hosmer–Lemeshow test was used to examine the goodness‐of‐fit of the final model, and a *P*‐value >0.05 indicated an acceptable fitness. All the analyses were performed using SPSS 19.0 (SPSS, Chicago, Illinois, USA).

## Results

### *General Characteristics*

**Figure 1:**
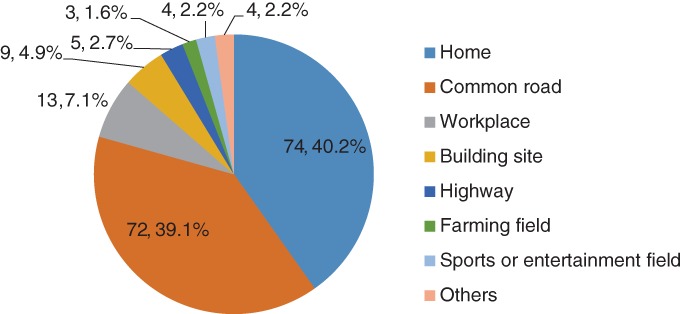
Places where low‐energy fracture occurred in 2014. Home and common road (other than high way) were the most common places that low‐energy upper extremity fractures occurred, representing 40.2% and 39.1%, respectively.

Of the 184 patients with 185 cases of low‐energy fractures, there were 44 men and 140 women; the average age at which fractures occurred was 63.1 ± 8.8 years (median, 62 years; range, 50–87 years). Home was the most common place where upper extremity fractures occurred, followed by the common road (other than high way) (39.1%) and workplace (7.1%) (Fig. 1). In total, there were 29 humerus fractures (proximal, 19; shaft, 8; distal, 2) and 155 radius (ulna) fractures (proximal, 7; shaft, 31; distal, 118).

### *Incidence of Low‐energy Fracture by Gender*

**Figure 2:**
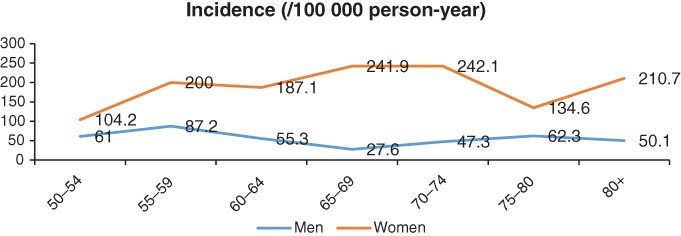
The trend of the incidence rate of low‐energy upper fractures with aging, in men and women. There was an obvious one peak in women, at the age of 55–59 years old, and a valley at the age of 75–80 years old; however, for men, the trend line is almost flat, without great fluctuation.

The overall incidence rate of low‐energy upper extremity fractures was 119.4 (102.2–136.6) per 100 000 persons in year 2014, with 57.4 (40.4–74.3) and 180.9 (150.9–210.8) per 100 000 person‐years in men and women, respectively (Table 1). The results of a trend test showed a significant increasing trend in the incidence rate of low‐energy upper extremity fractures with age in women (*P =* 0.041), but not in men (*P* = 0.369) (Table 1 and Fig. 2). In men, individuals aged 55–59 years had the highest incidence rate (87.2/100 000 person‐year), followed by those of 50–54 years (61/100 000 person‐year) and 75–79 years (62.3/100 000 person‐year). In contrast, women of 65–69, 70–74, and 80+ years had the highest incidence rates. Women living in the eastern region of China had the highest incidence rate of low‐energy upper extremity fractures, and the difference in incidences among the three regions was significant statistically (*P* = 0.004) but non‐significant for men (*P* = 0.371). For other variables, there were no significant differences among the respective subgroups, such as ethnicity, residency category, education, and occupation. Detailed information is presented in Table 1.

**Table 1:** National incidence of low‐energy upper limb fractures (cases/100 000 person‐years) in China by demographic, socioeconomic, and geographic factors in 2014

	Sample size	Incidence (cases/100 000 person‐years) (95% confidence interval)		
		Men	Women	Total
Individuals	154 099	57.4 (40.4–74.3)	180.9 (150.9–210.8)	119.4 (102.2–136.6)
Age (years)				
50–54	38 849	61 (26.5–95.5)	104.2 (58.6–149.9)	82.4 (53.8–110.9)
55–59	26 114	87.2 (35.7–138.7)	200 (124.6–275.3)	145.5 (99.3–191.7)
60–64	32 854	55.3 (19.2–91.4)	187.1 (121.3–252.9)	121.8 (84–159.5)
65–69	22 032	27.6 (0–55.2)	241.9 (150.8–333)	136.2 (87.5–184.9)
70–74	16 713	47.3 (1–93.7)	242.1 (136.1–348)	143.6 (86.2–201)
75–79	9275	62.3 (0–124.6)	134.6 (27–242.3)	97 (33.7–160.4)
80+	8262	50.1 (0–100.2)	210.7 (73.2–348.3)	133.1 (54.5–211.8)
*P*‐value for trend test		0.369	0.041*	0.183
Ethnicity				
Han nationality	144 433	59.8 (42–77.7)	177.8 (147.1–208.4)	119.1 (101.3–136.9)
Other nationalities	9666	20.7 (0–41.3)	227.3 (93.1–361.4)	124.1 (53.9–194.3)
*P*‐value for difference test		0.272	0.432	0.889
Urbanization				
Rural area	61 294	62 (34.1–89.8)	202.4 (152.1–252.8)	132.1 (103.4–160.9)
Urban area	92 805	54.3 (33–75.6)	166.7 (129.7–203.7)	111 (89.6–132.4)
*P‐*value for difference test		0.666	0.253	0.239
Region				
East	70 518	62.1 (36.2–88.1)	202.3 (155.3–249.3)	131.9 (105.1–158.7)
Central	30 224	73.9 (30.3–117.6)	247.6 (169–326.2)	162.1 (116.8–207.5)
West	53 357	41.7 (17.1–66.3)	115 (74.5–155.4)	78.7 (54.9–102.5)
*P‐*value for difference test		0.371	0.004*	0.002*
Education				
Illiterate	52 109	50 (21.7–78.3)	167.2 (119.5–215)	113.2 (84.3–142.1)
Primary school	54 373	63.8 (34.4–93.3)	240.6 (181.3–300)	149 (116.6–181.4)
Junior high school	42 761	46.8 (17.8–75.9)	126.1 (78.6–173.7)	86.5 (58.7–114.4)
Senior high school or above	4856	127.5 (2.6–252.4)	174.5 (0–359)	144.2 (37.4–250.9)
*P‐*value for trend test		0.495	0.421	0.470
Occupation				
Office worker	11 203	88.4 (17.7–159.2)	45.3 (0–90.6)	71.4 (21.9–120.9)
Farmer	57 412	47.8 (21.8–73.8)	182 (133.9–230)	118.4 (90.3–146.6)
Manual worker	29 396	50.8 (17.6–84)	162.6 (89.5–235.7)	95.3 (60–130.5)
Retired	30 357	60.6 (21–100.2)	225.6 (150.9–300.2)	144.9 (102.1–187.7)
Unemployed	17 505	29.6 (0–59.2)	214 (126.6–301.4)	142.8 (86.9–198.8)
Other	8226	146.8 (18.2–275.5)	124.5 (24.9–224)	133.7 (54.8–212.7)
*P‐*value for difference test		0.823	0.159	0.286

* Statistically significant.

### *Incidence of Low‐energy Fractures by Site*

Table 2 presents the incidence rates of humerus and radius (ulna) fractures for each fracture site (proximal, shaft, and distal). The total incidence of humerus and radius (ulna) fractures was 18.8 (12–25.7) and 100.6 (84.8–116.4) per 100 000 person‐years, respectively. In the respective subgroups, the incidence rate of proximal humerus fractures (18.8 per 100 000 person‐years) and distal radius fractures (100.6 person‐years) was highest.

**Table 2:** National incidence of low‐energy upper limb fractures by site (proximal, shaft, distal)

Item	Incidence rate per 100 0000 population (95% confidence interval)		
	Men	Women	Total
Humerus	6.5 (0.8–12.2)	31 (18.6–43.4)	18.8 (12–25.7)
Proximal	3.9 (0–7.8)	20.7 (10.5–30.8)	12.3 (6.8–17.9)
Shaft	2.6 (0–5.2)	7.8 (1.5–14)	5.2 (1.6–8.8)
Distal	0	2.6 (0–5.2)	1.3 (0–2.6)
Radius and ulna	50.9 (34.9–66.8)	149.8 (122.6–177.1)	100.6 (84.8–116.4)
Proximal	2.6 (0–5.2)	5.2 (0.1–10.2)	3.9 (0.8–7)
Shaft	13 (5–21.1)	25.8 (14.5–37.2)	19.5 (12.5–26.4)
Distal	35.2 (21.9–48.5)	118.8 (94.6–143.1)	77.2 (63.4–91.1)

### *Multivariate Analysis*

In men, alcohol consumption, residence at ≥2nd floor without an elevator, sleep duration<7 h/day, and history of past fracture were identified as significant risks factors associated with upper extremity fractures (Table 3). The Hosmer–Lemeshow test demonstrated the adequate fitness (*X* ^*2*^ = 7.968, *P* = 0.124).

**Table 3:** Results of multivariate logistic regression of risk factors for low‐energy fractures: upper extremity in men and women

Variables	Odds ration	95% confidence interval		*P*
		Lower limit	Upper limit	
Men				
Latitude zone				
20°–29.9°	Reference	–	–	–
30°–39.9°	0.52	0.27	1.01	0.054
40°–49.9°	1.50	0.67	3.39	0.327
Alcohol consumption	2.12	1.11	4.06	0.024*
Residence				
Ground floor	Reference	–	–	–
≥2nd floor without elevator	2.86	1.16	7.06	0.023*
≥2nd floor with elevator	1.30	0.67	2.51	0.438
Sleep duration <7 h/day	2.77	1.42	5.37	0.003*
History of previous fracture	3.10	1.21	7.93	0.018*
Women				
Body mass index				
18.5–23.9	Reference	–	–	–
24.0–27.9	0.93	0.37	2.31	0.872
≥28.0	1.86	1.31	2.66	0.001*
≤18.5	1.41	0.76	2.62	0.282
Region				
East	Reference	–	–	–
Central	1.53	1.01	2.31	0.045
West	0.57	0.37	0.88	0.011*
Latitude zone				
20°–29.9°	Reference	–	–	–
30°–39.9°	1.09	0.73	1.62	0.685
40°–49.9°	1.79	1.02	3.14	0.044*
Alcohol consumption	2.40	1.58	3.63	<0.001*
Every birth increased	1.45	1.15	1.83	0.002*
Sleep time < 7 h/day	2.21	1.53	3.20	<0.001*
History of previous fracture	2.70	1.52	4.80	0.001*

* Statistically significant.

In women, obesity (BMI ≥ 28.0 kg/m^2^), living in the central region, living at a higher latitude (40°–49.9°N), alcohol consumption, more births, sleep duration <7 h/day, and history of past fracture were identified to be associated with increased risk of upper extremity fractures. Living in the western region was a protective factor for low‐energy fractures (OR, 0.57; 95%CI, 0.37–0.88) (Table 3). The Hosmer–Lemeshow test demonstrated the adequate fitness (*X* ^*2*^ = 3.135, *P* = 0.926).

## Discussion

In this study, using data from the CNFS database, we have assessed the epidemiologic characteristics of low‐energy upper extremity fractures in middle‐aged and elderly men and women. The overall incidence of upper extremity fractures was 119.4/100 000 persons in 2014. Women had an approximately three times the risk of upper extremity fractures as men (150.9 *vs* 57.4 per 100 000 persons). Approximately 80% of all the fractures occurred at home and on the common road (other than high way), which emphasized the importance of the primary preventive measures. We observed a significant increasing trend in the incidence of low‐energy fractures with increasing age in women, but not in men. After adjustment for confounders, alcohol consumption, residence at ≥2nd floor without an elevator, sleep duration <7h/d, and history of past fracture were identified as significant risk factors for low‐energy fractures in men. In women, obesity, living in the central region, living at a higher latitude (40°–49.9°N), alcohol consumption, more births, sleep duration <7 h/day, and history of past fracture were identified as significant risk factors for low‐energy fractures.

Research on the epidemiology of upper extremity fractures from population‐based questionnaire surveys is scarce due to the substantial costs and low practical feasibility of face‐to‐face interviews with participants. Most research has focused on a single hospital, a certain region, or a subgroup of the population^13^, ^16^, ^17^, ^18^. In addition, differences in geographical location, socioeconomic development, culture practices, and individual lifestyles^19^, ^20^ contributed to different rates of incidence of fractures. In Denmark, Abrahamsen *et al.* 18 evaluated forearm fractures in adults above 50 years using national individual patient data on inpatients and outpatients, and reported the incidence of 278 and 1110 per 100 000 patient‐years for men and women. In the USA, data from Nationwide Emergency Department Sample (NEDS) showed that the incidence of humerus fractures was 122/100 000 person‐years in populations of all ages, and the incidence of proximal humerus fractures was 73 and 217 per 100 000 person‐years in men and women above 50 years^21^. In Hungary, the incidence of proximal humerus fractures was reported as 47 and 84 per 100 000 person‐years in men and women ≥50 years^22^. In contrast, in some Asian countries or regions, the incidence of upper extremity fractures was slightly lower. In Japan, Sakuma *et al.* reported that the incidence of proximal humerus and distal radius fractures was 37.1 and 108.6 per 100 000 person‐years, respectively in Sado City inhabitants above 50 years of age, both of which values were comparable to ours. Similarly, in Chinese Taiwan, researchers reported that the incidence of distal fractures was 80.6–100 and 123–189 per 100 000 person‐years for men and women, from 2006 to 2007^17^. The reported incidence of upper extremity fractures in this study was much lower than that of Western developed countries but comparable to that of East Asian countries or regions. The underlying mechanism might be related to the less frequent falls^23^ and relatively low prevalence of osteopenia and osteoporosis^24^ in East Asian populations. However, low‐energy upper extremity fractures undoubtedly constitute a major public health issue in modern China, due to the huge number of middle‐aged and elderly individuals.

We observed a significant increasing trend in the incidence of low‐energy fractures with increasing age in women, but not in men. This discrepancy in sex was largely explained by the physiology structure and estrogen levels, social roles, and lifestyles^25^, ^26^, ^27^, ^28^. Following menopause, women produce less estrogen, which results in the reduction of bone mass and bone mass density (BMD)^27^. Simultaneously, menopause‐related reduced sleep duration, dementia or climacteric syndromes would exert negative effects on bone metabolism or increase the risk of falls^27^. In addition, with age, underlying chronic diseases, such as diabetes and cardiovascular and cerebrovascular diseases, undoubtedly are associated with increased risk of osteoporosis and related fractures. Therefore, relative to male individuals, bone mass or BMD in women had to suffer more threats from advanced age, reduced estrogen level and others.

Alcohol consumption and insufficient daily sleep duration (<7 h/day) were identified as modifiable risk factors, for both men and women. Clark *et al.* 25 suggest that excess alcohol consumption increases the risk of fractures through metabolic effects, *via* alcohol‐related falls, and having a more hazardous lifestyle generally. Scholes *et al.* 26 reported that consuming more than 6–8 units of alcohol for women or men ≥55 years significantly increased the risk of fracture, by 1.65–2.07 times. Stone *et al.* 27 and Holmberg^28^ report on the relationship between insufficient sleep and the risk of fracture, in men and women, and show that insufficient sleep is related to increased risk of frequent falls. Accordingly, health policies that focus on decreasing alcohol consumption and encouraging individuals to improve their quality and duration of sleep should be implemented in China to reduce risk of fracture.

In men, another modifiable factor was living place and results showed that men who reside on the 2nd floor or higher without an elevator had a 2.86 times increased risk of upper extremity fractures and this might be related to the decreased muscle strength, poorer muscle coordination, and impaired vision in elderly individuals^23^. Therefore, the significance of moving to the ground floor or to a building equipped with an elevator should be emphasized. In women, another modifiable risk factor was obesity, which was shown to increase the risk of upper extremity fractures by 86%, compared to the normal BMI (18.5–23.9 kg/m^2^). This result was consistent with that of a previous meta‐analysis^29^, which suggested that being overweight increased the risk of upper arm fractures, while being underweight increased the risk of hip fractures. Therefore, maintaining a healthy body weight with a normal BMI is of clear significance in the prevention of both upper and lower extremity fractures. Several other non‐modifiable risk factors have been identified to be associated with upper extremity fractures in women, including number of births and geographic position. This might be related to the more rapid BMD decrease from the peak value at 32–34 years with more births, the intense pace of life, and more frequent exposure to the hazardous lifestyles in the eastern region, and less sunshine duration and intensity. For example, Ladizesky *et al.* 30 suggested that synthesis of vitamin D is absent during the 3 or 4 months of winter in higher latitude regions, where sunlight intensity and duration were relatively less than in lower latitude regions.

### *Strengths and Limitations*

The main strengths of the current study included the stratified multistage cluster randomized sampling method for recruiting subjects, the face‐to‐face interviews for data collection, and the adjustment for numerous important covariates. In addition, “double” confirmation of fracture cases through patients’ self‐reports and clinical or radiographic data increased the accuracy and precision of diagnosis.

However, there are some potential limitations that should be considered. First, there was some recall bias due to the retrospective design of the study. Second, there remained some residual confounding, because other factors (diabetes, hyperthyroidism, and past use of hormone therapy) that may have affected BMD or propensity to fall were not able to be adjusted for in the multivariate analysis model. The qualitative rather than quantitative definition of smoking or alcohol consumption was used, which might affect the precision of the statistical results when identifying the related risk factors. Third, the study could not capture information about fracture cases in which the individual died, or about fractures (hip fracture) that were associated with a high 1‐year mortality rate. Overall, the incidence rate of low‐energy upper extremity fractures was underestimated.

This study provided detailed epidemiologic information about low‐energy upper extremity fractures, including population‐based incidence, place of occurrence, and the associated risk factors, which could be used as clinical evidence for health‐care planning and preventive efforts in China, as elsewhere. Accordingly, health policies that focus on decreasing alcohol consumption and encouraging individuals to improve their quality and duration sleep should be implemented in China. The significance of moving to the ground floor or to a building equipped with an elevator for men, and maintaining a healthy body weight for women should be emphasized to prevent upper extremity fractures.
